# Health effects of single motherhood on children in sub-Saharan Africa: a cross-sectional study

**DOI:** 10.1186/1471-2458-14-1145

**Published:** 2014-11-05

**Authors:** Lorretta FC Ntoimo, Clifford O Odimegwu

**Affiliations:** Demography and Population Studies Programme, Schools of Public Health and Social Sciences, University of the Witwatersrand, Johannesburg, South Africa; Department of Demography and Social Statistics, Federal University Oye-Ekiti, Oye-Ekiti, Ekiti State Nigeria

**Keywords:** Single motherhood, Stunting, Under-5 mortality, Public health interventions, Sub-Saharan Africa

## Abstract

**Background:**

Although progress has been made toward reducing child morbidity and mortality globally, a large proportion of children in sub-Saharan Africa still die before age five and many suffer chronic malnutrition. This study investigated the influence of single motherhood on stunting and under-5 mortality in Cameroon, Nigeria and Democratic Republic of the Congo (DRC). Particular attention was paid to the influence of mother’s economic resources, parental care and health behaviour on the difference in children’s health in single and two-parent families.

**Methods:**

Data were obtained from most recent Demographic and Health Surveys in Cameroon (2011), Nigeria (2008) and DRC (2007). The sample included women aged 15–49 years old and their under-5 children 11,748 in Cameroon, 28,100 in Nigeria, and 8,999 in DRC. Logistic regression and Cox proportional hazard analysis were used to estimate stunting and under-5 mortality, respectively.

**Results:**

The result showed that compared with children whose mothers were in union, children of single mothers who were not widows were more likely to be stunted (OR 1.79 p < 0.01 in Cameroon and 1.69 p < .01 in DRC). Economic resources and parental care significantly influenced the higher odds of stunting in single mother households in Cameroon and DRC. Relative to children of mothers in union, the risk of under-5 mortality in single mother families was higher in the three countries (HR 1.40 p < .05 in Cameroon, 1.27 p < 0.10 in DRC, 1.55 p < .01 in Nigeria). Economic resources, parental care and health behaviour accounted for the difference in Nigeria and Cameroon; in DRC, only economic resources had marginal influence.

**Conclusions:**

Single motherhood is a risk factor for children’s nutritional status and chances of survival before age 5 years in sub-Saharan Africa. To achieve improved reduction in children’s exposure to stunting and under-5 mortality, there is the need for public health interventions targeted at single mother households in sub-Saharan Africa.

## Background

Although progress has been made toward reducing child morbidity and mortality globally, many children under five years in developing countries are still vulnerable to preventable illness and die of causes related to malnutrition [1–5]. Specifically, child malnutrition and survival have remained a major public health challenge in sub-Saharan Africa. In 2011, the region had the highest under-5 mortality in the world (109 per 1000 live births), the highest prevalence of stunting (40%) and the second highest rate of wasting (9%) after South East Asia [6]. The second largest number of stunted children in the world after India is in Nigeria where over 11 million children under five years are stunted. Out of 6.6 million under-5 deaths in 2012, about half died in India, Nigeria, DRC and China; and Nigeria accounts for 13% of these deaths [7]. Bryce et al. [8] noted that poor child health and survival outcomes in developing countries remain because many of the children and mothers who need public health interventions are not reached.

Sub-Saharan Africa is experiencing steady growth in out-of-wedlock motherhood, marital instability, and widowhood exacerbated by wars and HIV/AIDS pandemic, which has resulted in a large number of single mother families in the region [9–16]. For instance, in Cameroon, the population of married women who were separated, divorced or had absent spouses increased from 78,060 in 1976 to 129,000 in 2005 (65%) and widows increased from 299,690 to 475,930 (58%) within the same period [17]. In Nigeria, close to one million women aged 10–85 years old were either divorced or separated women in 2006, and 1.7 million were widowed [18]. In DRC, divorce and separation increased by more than 50% in all age groups of women aged 15–49 years, between 1984 and 2007; for instance, divorced and separated women aged 20–24 years increased from 3.4% in 1984 to 7.5% in 2007 [19]. The proliferation of single mother families indicates that many children in sub-Saharan Africa are born and reared in single mother families. Studies in other regions, particularly in the West, associated single motherhood with many adverse effects on children’s well-being, including poor nutritional status and lower chances for survival between age 0–59 months [20, 21]. Given the high poverty levels in many sub-Saharan African countries, and gender gap in education and high-wage employment, many children of single mothers are likely to be at risk of malnutrition and under-5 mortality.

Although studies in sub-Saharan Africa have examined the influence of family structure and characteristics on child nutritional status and survival [10, 22–24], few paid specific attention to single mother households. Hence, little is known in the region about the influence of single mother households on child nutritional status and survival, and the particular factors that account for differences between single mother families and two-parent households. This study examined the association between single motherhood and child health, indicated by stunting and under-5 mortality, in three sub-Saharan African countries: Cameroon, Nigeria and Democratic Republic of the Congo (DRC). The study has two objectives. The first is to examine the influence of single motherhood on children’s health, marked by stunting and under-5 mortality. The second is to investigate the independent and aggregate effect of mother’s economic resource, parental resource and health behaviour on the relationship between single motherhood and children’s health. Thomson and McLanahan [25] stress the need for cross-national research on the link between family structure and child well-being. The analytical framework proposed by Mosley and Chen [26], that integrates growth faltering and under-5 mortality as indicator of health status in a population, underscores the importance of studying stunting and under-5 mortality together.

Under-5 mortality is a pivotal indicator of child well-being and human development of a country [27], and height-for-age is the best predictor of human capital [28, 29]. Poor health in childhood affects productivity in adult life and reduces overall economic productivity in a country [30]. Specifically, stunted children are more likely to have poor cognition, lower educational performance and economic status, poor health in adulthood and a higher likelihood of mortality, and the effects are inter-generationally transmitted [28, 30–32]. According to the World Bank, productivity loss to individuals as a result of childhood malnutrition is more than 10% of life-time earnings and countries lose between 2 to 3 percent of their gross domestic product (GDP) to malnutrition [30]. Child health and survival are, thus, consequential for human and economic development in sub-Saharan African countries [33, 34]. This study contributes to the achievement of the World Health Assembly’s new goal to reduce the number of stunted children worldwide by 40 percent in 2025; the United Nations Secretary General’s Zero Hunger programme; and reduction of under-5 mortality.

### Single motherhood and children’s health

Relative to children raised in two-parent households, many studies, particularly in Western countries, associated single motherhood with poorer physical and mental health, and higher risk of mortality for children [25, 35–43]. On the other hand, a few studies have also found that in spite of lower earnings, children in female-headed households experience better or equal advantage in nutrition compared to those in male-headed or two-parent households [44, 45]. This association is explained by mother’s higher likelihood to invest in child welfare. The relative advantage of children in two-parent families in well-being is associated more with stable unions, especially of two biological parents [46, 47]. Living in step-parent households has adverse effects on child well-being [48]. Conflictual marriages lead to as much disadvantage in child welfare as disrupted unions. In some cases, single parenting by a mother after separation is more advantageous for children [46, 49, 50], particularly if the father engages in high anti-social behaviour [51].

#### Single motherhood, child nutritional status and mortality

A study in Peru showed that children who did not see their fathers daily and weekly, particularly during childhood, because of non-marriage to their mothers, divorce, separation or father’s migration were more likely to be stunted, but the effect was attenuated by urban residence, wealthiest household and food secure environment [52]. Bronte-Tinkew and DeJong [53] found that the likelihood of stunting among Jamaican children was 4.89 times higher in single-parent households than in two-parent families. Although the authors did not disaggregate single parents by sex, single parents are known to be more of mothers than fathers. An examination of family influence on stunting and infant mortality across 42 countries in Latin America, Africa and Asia showed that female-headed households were insignificantly associated with higher likelihood of mortality and significant lower odds of stunting [54]. The risk of stunting in that study was about 25% lower in female-headed households. With the advantage of fewer children, single mothers who can afford money and time resource necessary for child well-being [21] are likely to have children who are not stunted. However, because the authors used female household heads, the effect of single motherhood on stunting and under-5 mortality in their study is not certain. This finding indicates the need to separate female household heads who are single mothers from those who have partners that live elsewhere, and to disaggregate single mothers by pathway.

In sub-Saharan Africa, few studies paid attention to mother’s marital status in their examination of children’s nutritional status [22, 23, 55]; others focused on female-headed households [56–58]. Gurmu and Etana [55] found that single-parent nuclear family as a variable significantly increased the likelihood of stunting in Ethiopia, but sex of household head as a variable was not significant; hence, the study did not show whether the single-parent family was single mother or father. Gage [23] found that, in Kenya children’s likelihood of stunting had no significant association with mother’s marital status.

Few studies on under-5 mortality in sub-Saharan Africa examined family structure as key independent variable, with specific attention to single motherhood. Omariba and Boyle [59] examined variations in the relationship between under-5 mortality and family structure across 22 sub-Saharan African countries and the effects over time, with specific emphasis on polygyny. Children in single mother households were 16.3% more likely to die than those in monogamous union, and the relationship was found constant over time. They compared married (monogamous/polygynous) and single mother families, but they did not compare single mother families by single mother families. In Clark and Hamplova [10], relative to continuously married mothers in 11 sub-Saharan African countries, children of single mothers (premarital and divorced) were found to be more likely to die before age 5 years. Coefficients for widows were not significant except in one country. Their study was silent on women who were separated (not living together); it is assumed they were included into the divorced category. It is also not quite clear how the study handled currently never married mothers in the countries without monthly marriage history because their definition of never married mothers was mothers who had births before marriage. The current study extends these past studies by not only examining influence of single motherhood, disaggregated into widows and other single mothers, on stunting and under-5 mortality; it also examined the factors that account for any difference in outcome between single mother and two-parent households.

#### Theoretical perspectives

Explanation of the adverse effects of single motherhood on children’s health, survival and well-being in general is usually anchored on economic resource, parental resource or socialisation, dilution, and selection effect models. The economic resource model posits that child well-being is closely linked to economic resources available in a family, and that relative to two-parents households, single mothers have less economic resources [21]. This perspective is supported by other studies that examined the effect of household resources on children’s health. For instance, Victora et al. [60] strongly showed that chronic and acute under-nutrition, and under-5 mortality are primarily associated with inequalities in financial and material resources between households and nations. The economic resource model has been confirmed in studies that examined the link between single motherhood and adverse child health and survival [43, 61, 62]. The relative advantage of children in two-parent families in economic resources is however, dependent on if the parents are gainfully employed; optimally committed to children’s well-being, and the commitment is expressed in substantial cash contribution to children’s nutrition and health care [22, 52]. Economic deprivation in single mother households may be attenuated by kin support [63–65]. However, support from kin is influenced by cultural norms about the appropriateness of single motherhood by other pathways except widowhood [23]. Increasing urbanisation and nucleation of families are also reducing the extent and quantity of support single mothers get from near relatives and friends [66].

Furthermore, the effects of single motherhood on child well-being vary by personal socioeconomic characteristics and type of single motherhood. Bock [67] studied children of single mothers by choice in the USA who were highly educated and had stable employment. The author found that the children did well and were comparable to children in two-parent households. This suggests that when single mothers are highly educated and their lone motherhood is deliberate and planned, children are likely to experience no unusual health outcome. Several studies in sub-Saharan Africa found maternal education, reading skill, and cash earnings particularly significant for improved child health and survival [55, 59, 68–74]. Although economic resources available to a mother are vital for child nutritional status and survival, in some cases malnutrition persists in spite of increase in income and households wealth status [33]. A possible explanation may be that educated and employed mothers who leave their preschool children for domestic servants, relatives or nannies during working hours have little or no control over the quantity of food a child gets during those hours [75, 76].

Studies that explain the relative disadvantage of children in single mother families from the perspective of parental resource or socialisation argue that the absence of one parent in a family reduces time resource available for monitoring and supervision of children. Combining work and parenting not only affects the time a single mother has for child care, but it also introduces stress that negatively affects her psychological well-being and parenting effectiveness [61]. Consistent with the socialisation model, Thomson, McLanahan and Curtin [77] found that single parents exercised less control and supervision on their children than married parents. However, when close kin, such as grandmothers, are available to help in child care, the stress reduces, parenting becomes more effective, and child health and survival outcome is better [63–65]. Nelson [78] demonstrates from her study with White rural single mothers in the USA that single mothers maintain effectiveness in parenting even when other kin assist in care-giving. On the contrary, Reyes et al. [79] found, in a study conducted in Mexico, that children who were not taken care of exclusively by their mothers had higher risk of linear growth retardation. From the perspective of the socialisation model, the current study argues that children of single mothers in sub-Saharan Africa are unlikely to have many health disadvantages if the mother is not young, works from home, have older children, and has kin support.

The resource dilution model asserts that large family size reduces the quantity and quality of material, time, care and attention that flow from parents to children, which result in poorer outcome in children [53, 80, 81]. Large sibsize has been found to increase the risk of stunting [71] and under-5 mortality [82], in some sub-Saharan African countries. However, Houle et al. [82] found that presence of adult relatives in a household resulted in lower odds of under-5 mortality. Given that single mothers are likely to have fewer children than married mothers, if commitment to their children’s welfare is optimal, such children will have less health disadvantage, especially if the mother is not an adolescent, has sufficient time for parental supervision and gets support from kin and friends.

An aspect of household production of health is incorporated to further explain any variation in child health and survival outcome between single and married mothers and among groups of single mothers. Household production of health argues that health behaviour of household members has more direct influence on individual health outcome than availability of health facilities and services [83]. In particular, mothers’ health behaviour, such as antenatal care, duration of breastfeeding and birth interval, has direct consequence for children’s health and survival. Previous studies in sub-Saharan Africa identified duration of breastfeeding, birth interval, place of delivery, antenatal care, mother’s nutritional status during pregnancy, among others, as significantly related to stunting, infant and child mortality [56, 59, 82, 84]. Noteworthy is the fact that the culture of long duration of breastfeeding in sub-Saharan Africa [85] is being threatened by increasing female participation in the labour force [86, 87]. Single mothers in the USA, for instance, have been found to work longer hours than married mothers [21]. It is expected that if the same applies to single mothers in sub-Saharan Africa, their health behaviour in terms of breastfeeding will be poorer than that of married mothers. With regard to other health behaviour, single mothers should have more autonomy and less traditional views about health care than married mothers; which should be an advantage for their children in terms of health in general [69], and stunting in particular [88], especially if they are educated and have sustainable livelihood.

## Methods

This study is based on analysis of existing data set obtained from Demographic and Health Surveys (DHS) conducted in Nigeria (2008), Cameroon (2011) and DRC (2007). Children’s recode was used. The study population comprised a nationally representative sample of women aged 15–49 years who had children under five years old. The weighted sample of mothers and children were 11,748 in Cameroon; 28,100 in Nigeria; and 8,999 in DRC. Permission to use the data was obtained from ICF Macro Inc. Details on the sampling procedure are available elsewhere (ICF International).

The estimated percentages of women who experienced single motherhood by any pathway in Cameroon (2011), DRC (2007) and Nigeria (2008) were 34%, 27% and 16%, respectively. Although prevalence of single motherhood in Nigeria is below 20 percent, the country has the highest number of stunted children in the region and very high under-5 mortality, 124 per 1000 live births (UNICEF, 2013). Of the countries in Middle Africa with the high percentage of women who experience single motherhood by any pathway, Democratic Republic of the Congo (DRC) has the highest prevalence of stunting (43%) and high under-5 mortality (168). Cameroon has high single motherhood (34%), under-5 mortality (127) and relatively high stunting prevalence (33%). In Cameroon, stunting is particularly highly prevalent and severe in the rural areas [89]. Results from countries with different proportions of women who are single mothers, high under-5 mortality and prevalence of stunting are expected to provide better insight on the influence of single motherhood on children’s nutritional status and chances for survival.

### Dependent variable

Proxies for child health were nutritional status and mortality. The measure for child nutritional status was linear growth retardation (stunting), and mortality was measured with under-5 mortality. DHS provided anthropometric data on stunting for children aged 0–59 months. Stunting was measured as the proportion of children whose height-for-age was less than minus 2 standard deviations below the World Health Organisation (WHO) height-for-age or length-for-age standard median. The values were categorised into two: those whose height-for-age were below -2sd were stunted, coded 1; others were not stunted, coded 0. Under-5 mortality was measured as the probability of death from age 0 to death of a child before fifth birthday. The children’s survival status, alive or dead, and age at death in months were pooled to create the dependent variable for survival analysis.

### Independent variables

Single mothers of interest were mothers whose current marital status was never married, widowed, divorced and separated. For the purpose of the multivariate analysis, the key independent variable was mother’s current marital status, disaggregated into married, widowed, and other single mothers. Other single mothers included the never married, divorced and separated. Married mothers were the reference category. Married was defined to include those who were in union, whether married or living together. Marital history in the dataset did not provide dates of marital disruption; therefore, incidence of malnutrition or mortality cannot strictly be said to have taken place after marital disruption. Using dataset for children aged 0–59 months at the time of the survey limited the children under focus to those born five years before the interview. If a woman’s marriage was disrupted before five years to the survey, the probability that she gave birth after the disruption is low. Thus, it is likely that divorced, separated and widowed mothers who had under-5 children at the time of the survey experienced disruption in their marriages within the five years preceding the survey. Lack of information on the timing of marital disruption would not introduce serious bias to the study.

The data used for this study did not provide information on income and household consumption. Therefore, index of household wealth, mother’s occupation, education, and place of residence were used as measures of economic resources. Household resources and occupation indicate access to financial and material resources; education is a non-material resource (human capital); whereas place of residence influences one’s social capital in form of social connections that can influence children’s well-being [90]. For instance, in rural areas, single mothers might have more access to close kin who provide assistance in parental care. On the other hand, rural residence increase risk of poor health for children owing to lower economic resources, earlier age at first child birth, and lack of access to modern medical facilities and services [91, 92]. Occupation was categorised into professionals/clerical workers, sales/services, agriculture, skilled and unskilled manual worker and others, such as domestic servants. Education was the highest level of education attained, and place of residence was rural and urban. Household wealth was measured as poorest, poorer, middle, richer and richest.

Measures of parental resource included work location, whether the mother’s place of work was located at home or away from home. This was used as an indication on how much time a mother spends with her children. Other measures were age at first birth (<= 19 and 20+ years), an indication on parenting effectiveness and number of surviving children (<=2, 3–4 and 5+) was a proxy for resource competition among children in a family and presence of older children. Presence of older siblings, particularly sisters, in some cases, enhance nutritional status for younger siblings; constraints of parental time and financial resources sometimes lead to better care for boys than girls [93, 94]. Size of household is also an indication of crowding, a risk factor for child health.

Mother’s health behaviour was marked with antenatal care visits (less than 4 and more than 4), duration of breastfeeding, preceding birth interval (<2 years and >2 years), place of delivery (home and private or public health facility), and body mass index was used as a proxy for mother’s nutritional status. DHS collected data on individual women’s body mass index. For the purpose of this study it was categorised into three: thin (<18.50 kg/m^2^), normal (18.50-24.99 kg/m^2^), and obese (>25.00 kg/m^2^). Malnourished mothers are more likely to have babies with low birth weight, lower quality and quantity of breast milk, and other poor health conditions that affect child health and survival [26, 95]. Duration of breastfeeding was categorised into three for stunting (never breastfed, still breastfeeding and ever breastfed), and two categories for under-5 mortality (never breastfed and ever breastfed). Duration of breastfeeding is closely associated with stunting and under-5 mortality [4, 6]. Previous studies in Nigeria, Cameroon and DRC link these behaviours to child nutritional status and mortality [96, 97].

### Control variables

Drawing on evidence in past research, variables that are likely to influence the relationship between single motherhood and child stunting and survival, economic resources, parental resource and health behaviour were controlled. This was to strengthen conclusions drawn from the study. To control the effects of partner’s resource advantage for married mothers, partner’s highest education and occupation were added. Religion and region of origin influence women’s socioeconomic status, parenting pattern, nutrition, and health-seeking behaviour. Region and ethnic origin influence patterns of under-5 mortality and malnutrition in sub-Saharan Africa [98]. Specifically, in Nigeria and DRC region influences child mortality and morbidity [92, 99]. In Cameroon, childhood feeding practices varies between Muslims and non-Muslims in the northern region [97]. Under-5 mortality is influenced by religious affiliation and cultural beliefs in Cameroon and Nigeria [100, 101]. Child characteristics that were controlled included birth weight (<2.5 kg and >2.5 kg), multiple or single birth, birth order, child’s sex, and experience of diarrhoea two weeks before the survey.

### Analytical approach

Five models were estimated using logistic regression and Cox Proportional Hazard model for stunting and under-5 mortality, respectively. The first model, which was the baseline, estimated stunting and under-5 mortality as a function of mother’s marital status. Models 2, 3 and 4 adjusted for economic resource, parental resource and health behaviour, respectively. Model 5 combined all the variables to examine the influence of single motherhood on children well-being, when all the variables are taken into consideration. In all the models, child and other background characteristics that are likely to influence the relationships were controlled. Logistic regression models are appropriate for estimating the log of the odds of a dichotomous dependent variable occurring as a function of a vector of the independent variables and an error term. Cox proportional hazard model was chosen because of its flexibility in handling censored observations while adjusting for explanatory variables. It generates relative risk and allows for no parametric assumptions [102]. In this study, children who were alive at the time of the survey were right censored, and those who died within the observation time were non-censored.

## Results

### Descriptive profile of the study population

Table 1 presents descriptive analysis of the study population (under-5 children and their mothers) in relation to selected variables. Results are presented in percentage. The values were weighted to adjust for over-sampling and under-sampling. Out of 3777 children in DRC who had height-for-age measures, 44.5% were stunted. In Cameroon, out of 5053 children who were measured for stunting, 32% were stunted. In Nigeria, 41% of 18.823 children measured were stunted. Under-5 mortality in percentage ranged from 9% in Cameroon to 11% in Nigeria and DRC. The majority of the children who died before age five died within the first month after birth in Nigeria and Cameroon, and within the first one year of life in DRC. The mean age of mothers in Nigeria and DRC was 29 years (sd 7.0 and 7.2 respectively), and 28 years (sd 6.7) in Cameroon. Among single mothers, there were more never married mothers in Nigeria and Cameroon, but those who were separated predominated in DRC.

**Table 1:** **Descriptive characteristics of the study population by selected variables**

Variable	Nigeria	Cameroon	DRC
	(n = 28,100)	(n = 11,748)	(n = 8,999)
Stunted	40.56	31.96	44.48
Child died	11.12	8.77	11.00
Marital Status			
Married	93.95	72.72	79.69
Living together	1.74	15.64	9.93
Never married	1.66	6.26	2.36
Widowed	1.11	1.25	1.22
Divorced	0.72	0.91	1.19
Separated	0.82	3.22	5.61
Maternal Education			
No education	46.52	28.59	23.52
Primary	23.21	39.48	41.90
Secondary	24.90	28.92	33.71
Higher	5.38	3.01	0.87
Place of residence			
Urban	29.75	42.22	38.78
Rural	70.25	57.78	61.22
Occupation			
Prof/clerical	3.67	1.81	2.12
Not working	30.38	25.79	21.96
Sales/services	37.38	24.33	17.69
Agriculture	17.35	33.68	54.65
Manual/others	11.22	14.39	3.58
Wealth Index			
Poorest	23.22	23.65	20.82
Poorer	22.76	21.64	22.61
Middle	19.28	20.00	20.71
Richer	17.80	19.01	20.49
Riches	16.94	15.70	15.37
Sibsize			
1-2	35.45	39.40	36.67
3-5	35.84	33.45	29.63
5+	28.71	27.14	33.70
Prenatal care			
No visits	40.04	14.62	12.95
<4 visits	10.59	22.54	38.34
>4 visits	49.37	62.84	48.72
Breastfeeding			
Never breastfed	4.27	4.58	6.00
Still breastfeeding	32.45	30.48	37.64
< 6 months	2.21	2.64	1.97
6-12 months	12.69	17.55	9.07
1-2 years	47.27	42.48	39.67
2+ years	1.11	2.27	5.65
Birth interval			
< 2 years	23.78	21.15	24.48
2+ years	76.22	78.85	74.52
Body Mass Index			
Normal (18.50-24.99)	66.22	61.70	74.09
Thin (<18.50)	11.26	7.54	13.80
Obese (>25.00)	22.52	30.76	12.11

A larger percentage of mothers of under-5 children in the three countries had primary or no education, no employment, worked in sales or service-related employment and agriculture. Many of those who were employed worked away from home (51% in Nigeria and 86% in DRC). The majority were from poor households. Except in Nigeria, the majority delivered their children in health facility (71% in DRC, 62% in Cameroon and 35% in Nigeria). Duration of breastfeeding for many of the respondents was between one and two years. Between 58 and 66 percentage had their first births before age 19 years, but birth spacing of more than two years was common among them. Many of the mothers had normal body mass index, and visited hospitals for prenatal care more than four times during pregnancy. However, the mothers differed in these health behaviour indicators. Kruskal-Wallis rank test of equality conducted on the health behaviour indicators, antenatal visits, breastfeeding, birth interval, and body mass index showed there was considerable significant difference in these health behaviours among mothers by marital status, in the three countries (result not shown).

### Single motherhood and stunting

Table 2 presents result of a two-tailed logistic regression on the influence of single motherhood on stunting; the independent and aggregate effect of economic resource, parental resource and health behaviour on the relative difference in stunting in single and married mothers households. Five models were fitted to examine the relationships. All models controlled the effects of child characteristics, partner’s education and occupation, and other maternal characteristics that influence child health and survival. Model 1 tested the effect of single motherhood. Relative to children of mothers in union, children in never married, divorced and separated single mother families had higher likelihood of being stunted in Cameroon and DRC (OR 1.79 and 1.69 p < 01), but the odds were insignificantly lower in Nigeria. Adjusting for economic resource in model 2, the higher odds in other single mother families relative to mothers in union remained significant, but reduced by less than 10% in Cameroon (OR 1.68 p < .05) and slightly in DRC (OR 1.68 p < .01). Model 3 tested the effect of parental resource variables. Children in single mother households, except widows, were significantly more likely to be stunted than those in two-parent households in Cameroon and DRC (OR1.78 p < .01 and 1.76 p < .01) respectively. Parental resource variables reduced the odds of stunting only slightly in Cameroon, but the likelihood in DRC increased. In model 4, the effect of maternal health behaviour was examined. The likelihood of stunting was lowered, but was insignificantly higher for other single mother’s children in Cameroon and DRC than children of mothers in union. In Nigeria, the result was still not significant. Model 5 measured the aggregate effect of economic resource, parental resource, and health behaviour on children’s likelihood of being stunted in single versus married-mother household. Children in single mother families in Cameroon and DRC were insignificantly more likely to be stunted than children in two-parent households; in Nigeria, the odds remained insignificantly lower for single mothers.

**Table 2:** **Logistic regression of mother's socioeconomic status, parental care and health behaviour on stunting**

Cameroon (2011)					
Models					
Marital status	Baseline	2	3	4	5
	OR/(CI)	OR(CI)	OR(CI)	OR(CI)	OR(CI)
In union (RC)					
Widowed	1.17	1.12	1.14	1.05	0.94
	(0.47-2.92)	(0.45-2.79)	(0.46-2.78)	(0.40-2.71)	(0.36-2.44)
Other single mothers	1.79**	1.68*	1.78**	1.36	1.23
	(1.17-2.74)	(1.09-2.60)	(1.16-2.73)	(0.84-2.21)	(0.74-2.04)
Constant	0.21***	0.37†	0.21***	0.41	1.07
	(0.10-0.41)	(0.11-1.19)	(0.11-0.42)	(0.10-1.67)	(0.14-7.80)
**Nigeria (2008)**					
**Models**					
**Marital status**	**Baseline**	**2**	**3**	**4**	**5**
	**OR/(CI)**	**OR(CI)**	**OR(CI)**	**OR(CI)**	**OR(CI)**
In union (RC)					
Widowed	0.87	0.85	0.90	0.72	0.70
	(0.62-1.23)	(0.61-1.18)	(0.61-1.32)	(0.47-1.11)	(0.46-1.07)
Other single mothers	0.87	0.85	0.97	0.77	0.74
	(0.61-1.23)	(0.61-1.19	(0.64-1.46)	(0.52-1.16)	(0.50-1.11)
Constant	0.30***	0.34***	0.33***	0.15***	0.15***
	(0.21-0.42)	(0.22-0.50)	(0.22-0.51)	(0.07-0.32)	(0.06-0.34)
**Congo DRC (2007)**					
**Models**					
**Marital status**	**Baseline**	**2**	**3**	**4**	**5**
	**OR/(CI)**	**OR(CI)**	**OR(CI)**	**OR(CI)**	**OR(CI)**
In union (RC)					
Widowed	0.63	0.65	0.75	0.46	0.47
	(0.28-1.43)	(0.28-1.51)	(0.30-1.84)	(0.17-1.23)	(0.17-1.32)
Other single mothers	1.69**	1.68**	1.76**	1.19	1.28
	(1.19-2.41)	(1.17-2.41)	(1.18-2.63)	(0.69-2.06)	(0.73-2.25)
Constant	0.09***	0.04***	0.07***	0.01***	0.00***
	(0.04-0.17)	(0.01-0.12)	(0.03-0.17)	(0.00-0.06)	(0.00-0.01)

Note: Model 2 adjusted for economic resource, model 3 parental resource, model 4 health behaviour. All models controlled for mother’s age, religious affiliation, region of origin, child characteristics (sex, age, birth weight, birth type – single or multiple, birth order) and partner’s characteristics – education and occupation.

CI: 95% confidence interval; *p < 0.05, **p < 0.01, ***p < 0.001 †p < 0.10. Work location was omitted in the final models because it was collinear with occupation category 1- not working. Model fit statistics: (Prob > F/chi2 for all the models in Cameroon, Nigeria and DRC, was significant p < .001.

### Single motherhood and under-5 mortality

The results of survival analysis are shown in Table 3. Five models were estimated to examine the influence of single motherhood on under-5 mortality; the effect of mother’s economic resource, parental resource, and health behaviour on the risk of under-5 mortality in single mother households. Underlying and proximate factors considered to influence chances of survival between age 0–59 months were controlled in all five models. Model 1 showed the influence of single motherhood on under-5 mortality, compared to mothers in union. Single mothers who were not widows in Cameroon were more likely to have children who died before age five (HR 1.40 p > 0.05). The hazard of under-5 mortality was also higher for children in other single mother households in Nigeria than in two-parent families (HR 1.55 p < .01). In DRC, children in other single mother households were marginally at a higher risk of mortality before age five years (HR 1.27 p < .10). Incorporating economic resource variables in model 2 slightly increased and retained the higher risk in other single mother families in all the countries (Cameroon HR 1.41 p < .05; Nigeria HR 1.58 p < .01; DRC HR 1.28 p < .10). In model 3, for parental resource, children of widows in Cameroon had 44% lower risk of child mortality before age five compared to those in two-parent households; whereas children in other single mother households were insignificantly more likely to die before age five than children of mothers in union. In Nigeria, parental resource marginally decreased the risk of under-5 mortality in other single mother families by 16% (HR 1.30 p < .10) relative to children in intact families. Model 4 adjusted for health behaviour, children in other single mother households in Cameroon were marginally more likely to experience under-5 mortality than those in two-parent households (HR 1.59 p < .10). In Nigeria, the risk of under-5 mortality was 74% higher for children of other single mothers (p < .01) compared to children of mothers in union. In both countries, health behaviour variables increased the risk of under-5 mortality by 14% and 12%. In the combined model 5, the risk of death was significantly higher for children in other single mother families only in Nigeria (HR 1.51 p < .01).

**Table 3:** **Hazard ratios on the relationship between mother's socioeconomic status, parental care, health behaviour and under-5 mortality**

Cameroon (2011)					
Models					
Marital status	Baseline	2	3	4	5
	OR/(CI)	OR(CI)	OR(CI)	OR(CI)	OR(CI)
In union (RC)					
Widowed	0.67	0.65	0.56†	0.72	0.52
	(0.34-1.31)	(0.33-1.25)	(0.29-1.09)	(0.28-1.85)	(0.19-1.40)
Other single mothers	1.40*	1.41*	1.19	1.59†	1.43
	(1.01-1.93)	(1.02-1.95)	(0.88-1.60)	(0.91-2.77)	(0.80-2.54)
**Nigeria (2008)**					
**Models**					
**Marital status**	**Baseline**	**2**	**3**	**4**	**5**
	**OR/(CI)**	**OR(CI)**	**OR(CI)**	**OR(CI)**	**OR(CI)**
In union (RC)					
Widowed	1.06	1.05	1.05	1.02	1.05
	(0.73-1.54)	(0.72-1.52)	(0.73-1.50)	(0.60-1.74)	(0.64-1.73)
Other single mothers	1.55**	1.58**	1.30†	1.74**	1.51**
	(1.18-2.05)	(1.21-2.08)	(0.96-1.75)	(1.22-2.49)	(1.11-2.06)
**Congo DRC (2007)**					
**Models**					
**Marital status**	**Baseline**	**2**	**3**	**4**	**5**
	**OR/(CI)**	**OR(CI)**	**OR(CI)**	**OR(CI)**	**OR(CI)**
In union (RC)					
Widowed	1.38	1.40	1.20	0.75	0.75
	(0.79-2.41)	(0.80-2.45)	(0.65-2.21)	(0.30-1.89)	(0.26-2.16)
Other single mothers	1.27†	1.28†	0.94	1.14	0.89
	(0.96-1.67)	(0.97-1.68)	(0.69-1.29)	(0.72-1.78)	(0.53-1.49)

Note: Model 2 adjusted for economic resource, model 3 parental resource, model 4 health behaviour. All models controlled for mother’s age, religious affiliation, region of origin, child characteristics (sex, birth weight, birth type – single or multiple, birth order) and partner’s characteristics – education and occupation. CI: 95% confidence interval; *p < 0.05, **p < 0.01, ***p < 0.001 †p < 0.10 Model fit statistics: (Prob > F/chi2 for all the models in Cameroon, Nigeria and DRC was significant p < .001.

## Discussion

The objective of the current study was to examine the influence of single motherhood on children’s health, marked by stunting and under-5 mortality, and to investigate the independent and aggregate effect of mother’s economic resource, parental resource, and health behaviour on the relationship between single motherhood and child health.

The results showed that single motherhood was positively associated with stunting in Cameroon and DRC, but not in Nigeria. The positive association in Cameroon and DRC confirmed previous studies [52, 53, 55]. The insignificant and negative relationship between single motherhood and stunting in Nigeria supports a past study in Kenya [23], where single motherhood was insignificantly related to stunting. The finding also relates to a study in Nigeria [56] that found stunting insignificantly related to female-headed households, and another study of 42 developing countries by Heaton et al. [54] where female household headship was negatively associated with stunting.

Economic resource variables mitigated the effects of single motherhood on stunting in Cameroon and DRC, but the association remained significantly positive. This result supports past studies that associated poorer child health outcome in single mother families with economic deprivation [43, 61, 62]. Parental resource variables slightly reduced the risk of stunting in Cameroon, but escalated it in DRC. The escalation in DRC is likely to be related to many children in a single mother family competing for few economic and time resources; in both models 3 and 5, larger sibsize was a positive significant predictor of stunting in DRC (result available on request). This supports the dilution model [80, 81] and a previous study in sub-Saharan Africa that associated large sibsize with stunting [71]. DRC is confronted with widespread poverty, and food insecurity exacerbated by war [92].

Findings on under-5 mortality indicate significant positive relationship between single motherhood and under-5 mortality in the three countries; thus supporting past studies [10, 59]. Mother’s economic resource in single mother families in the three countries had no mitigating effect on the higher likelihood of under-5 mortality in other single mother households. This result indicates that economic deprivation in single mother households in Cameroon, Nigeria and DRC poses more serious risk to child survival than may have been realised by policy makers. Less than 1% of single mothers in these countries had tertiary education which attracts higher wage. The majority of them had no employment, worked in the agriculture sector, most likely as subsistence farmers; in sales, most likely as petty traders. This result not only confirms applicability or economic resource model in the three countries, but it also stresses the precarious economic situation of women in sub-Saharan Africa [33, 103], particularly single mothers, and highlights the need to empower women for better child survival [27].

In Cameroon, widows exhibited better parenting effectiveness than mothers in union. Large family size in Cameroon reduced the likelihood of under-5 mortality; it is likely that the feedback effect of older siblings in form of sib-socialisation [71, 80] was stronger in widow households than for mothers in union. In Nigeria, parental resource or socialisation variables mitigated the effect of single motherhood on the hazard of under-5 mortality, although the risk was still higher than for children of mothers in union. In addition to the likelihood of the advantage of sib-socialisation, single mothers in Nigeria may have accessed more kin assistance in child care than single mothers in DRC and Cameroon. Past studies in sub-Saharan Africa showed the positive effect of kin support and assistance, especially grandmothers helping their daughters in child care, in reducing likelihood of under-5 mortality [63–65].

Health behaviour variables increased the risk of under-5 mortality in Cameroon and Nigeria, but insignificantly lowered the risk in DRC. This result affirms the argument that under-5 mortality is still high in spite of public health interventions because many of the children and mothers who need them are not reached [8]. For instance, the majority of respondents in Cameroon (49%), Nigeria (63%) and DRC (49%) had more than 4 antenatal care visits during pregnancy (Table 1), but the quality of care is unsatisfactory in many sub-Saharan African countries because of inadequate skilled or trained health professionals [6, 8]. Also, although many respondents breastfeed for between one and two years, few, according to UNICEF [6], breastfeed their children exclusively for the first 6 months of life, a health practice that is vital for child survival. Kruskal-Wallis test conducted in this study (result not shown) indicated that single mothers ranked lower than mothers in union in duration of breastfeeding. A study in Eastern Nigeria associated marital separation with shorter breast feeding duration of six months than for married mothers [87]. This is not unlikely to be associated with single, post-divorce and separation strain [104, 105] and the likelihood of single mothers to work away from home and for longer hours [21].

Consistently in all the models for stunting and mortality, the ratios for children of widows were insignificant except in Model 3 for parental resource in Cameroon where the hazard of under-5 mortality was lower for widows’ children. This is similar to findings by Clark and Hamplova [10] in 10 out of 11 sub-Saharan African countries. This result suggests that children of widows in these countries are likely to have more access to economic resources if their mothers inherited her husband’s assets. It is also likely that they receive more kin support than children of mother’s whose marital status is still subject to sanctions based on traditional norms of marriage and family [85].

This study confirms that economic and parental resources available to single mothers significantly account for the higher likelihood of stunting and under-5 mortality in that family form, relative to others [21, 61, 77]. The poor influence of maternal health behaviour on the likelihood of stunting and under-5 mortality highlights the need to improve strategies of intervention on maternal health behaviour to reach the most vulnerable groups of mothers and children, such as single mothers and their under-5 children. The advantage of partner support and check on health behaviour [106, 107] gives children of mothers in union advantage in nutritional status and chances for survival.

## Conclusion

Results from this study suggest that single motherhood poses a challenge to child’s health and survival chances, but the challenges can be minimised if never married, divorced and separated single mother families have access to more economic resources, improve their parental resource and health behaviour. The findings are strongly suggestive of the need for welfare benefits for single mothers of under-5 children in sub-Saharan African countries, where it is not existent. The study highlights the need for family-based public health interventions, particularly those targeted at single mother households. Although there is no one way to achieving healthy surviving children, interventions targeted at single mother households will go a long way in reducing stunting and under-5 mortality, specifically in the countries studied, and in sub-Saharan Africa at large.

In general, this study confirms the centrality of family in children’s nutritional status and chances for survival [21, 24, 26, 54]. The findings show that irrespective of father’s resources, mother’s economic and parental resources, and health behaviour are vital in achieving healthy surviving children [108]. The results underscore the imperativeness of more pragmatic, effective and sustainable programmes aimed at improving mother’s socioeconomic status, nutritional status, parenting effectiveness through accessible and affordable institutionalised day-care centres, promotion of maternal health behaviour and mass production of affordable nutritious food. More scholarly investigations are needed to explore the effects of single motherhood on other aspects of child health in sub-Saharan Africa. This study made use of cross-sectional data which limited its potential to make predictions that are causal. Also, lack of detailed marital history in the dataset did not allow analysis based on duration of single motherhood. The result on parental resource may have been influenced by the variables used in this study to measure parental resource. Inclusion of other measures, such as hours spent in child care; supervision; and person who cared for the child, mothers exclusively or other persons may yield better results.
